# Isolation and Characterization of Novel Phages Targeting Pathogenic *Klebsiella pneumoniae*

**DOI:** 10.3389/fcimb.2021.792305

**Published:** 2021-12-03

**Authors:** Na Li, Yigang Zeng, Rong Bao, Tongyu Zhu, Demeng Tan, Bijie Hu

**Affiliations:** ^1^ Zhongshan Hospital, Fudan University, Shanghai, China; ^2^ Shanghai Public Health Clinical Center, Fudan University, Shanghai, China

**Keywords:** bacteriophage, phage therapy, *Klebsiella pneumoniae*, phage-host interactions, multidrug resistance

## Abstract

*Klebsiella pneumoniae* is a dominant cause of community-acquired and nosocomial infections, specifically among immunocompromised individuals. The increasing occurrence of multidrug-resistant (MDR) isolates has significantly impacted the effectiveness of antimicrobial agents. As antibiotic resistance is becoming increasingly prevalent worldwide, the use of bacteriophages to treat pathogenic bacterial infections has recently gained attention. Elucidating the details of phage-bacteria interactions will provide insights into phage biology and the better development of phage therapy. In this study, a total of 22 K*. pneumoniae* isolates were assessed for their genetic and phenotypic relatedness by multi-locus sequence typing (MLST), endonuclease S1 nuclease pulsed-field gel electrophoresis (S1-PFGE), and *in vitro* antibiotic susceptibility testing. In addition, the beta-lactamase gene (*bla*_KPC_) was characterized to determine the spread and outbreak of *K. pneumoniae* carbapenemase (KPC)-producing enterobacterial pathogens. Using these ST11 carbapenem-resistant *K. pneumoniae* isolates, three phages (NL_ZS_1, NL_ZS_2, and NL_ZS_3) from the family of *Podoviridae* were isolated and characterized to evaluate the application of lytic phages against the MDR *K. pneumoniae* isolates. *In vitro* inhibition assays with three phages and *K. pneumoniae* strain ZS15 demonstrated the strong lytic potential of the phages, however, followed by the rapid growth of phage-resistant and phage-sensitive mutants, suggesting several anti-phage mechanisms had developed in the host populations. Together, this data adds more comprehensive knowledge to known phage biology and further emphasizes their complexity and future challenges to overcome prior to using phages for controlling this important MDR bacterium.

## Introduction

The ongoing explosion of the antimicrobial resistance crisis has been accelerated by the overuse and misuse of antibiotics, causing the emergence and increased prevalence of multidrug-resistant (MDR) bacteria (Ventola, 2015). *Klebsiella pneumoniae*, a Gram-negative *Enterobacterales*, can be found in the environment and has been associated with community-acquired infections (Pendleton et al., 2013). The increasingly common mechanism of carbapenem resistance in *K. pneumoniae* is mediated by extended-spectrum β-lactamases (ESBLs), which are able to break the β-lactam structure in the most commonly used antibiotics (Pitout et al., 1997). Thus, infections of KPC-producing bacteria are consistently associated with poor clinical outcomes and represent a significant and continued threat to the healthcare system. As a result, there is a strong need to find alternative clinical treatments to conventional antimicrobials.

Bacteriophages (phages for short) are the most common and diverse biological entities in the biosphere, playing an important role in promoting bacterial diversity, structuring community composition, and being a key driver in nutrient turnover (Suttle, 2005). Lytic phages infect and lyse bacterial hosts and are an attractive alternative towards combating the fast development of antibiotic-resistant *K. pneumoniae* (Sulakvelidze et al., 2001; Hung et al., 2011). The use of phages has gained interest over the past years; and phages that are specific for MDR bacterial pathogens, including *Pseudomonas aeruginosa*, *Enterococcus* spp., *Mycobacterium abscessus*, *Staphylococcus aureus*, *Acinetobacter baumannii*, and *Escherichia coli*, have been identified and used to treat bacterial infections (Chibani-Chennoufi et al., 2004b; Capparelli et al., 2007; McVay et al., 2007; Letkiewicz et al., 2009; Schooley et al., 2017; Abd El-Aziz et al., 2019; Dedrick et al., 2019; Cano et al., 2020). These studies demonstrated that lytic phages can be harnessed to prevent and eradicate pathogenic bacteria, and in some cases, alleviate current medical challenges posed by antibacterial resistance and thus further reduce mortalities. While previous applications of phage therapy have shown promising results, the technology is still in a stage of development due to rapid evolution dynamics between phages and their hosts (Chibani-Chennoufi et al., 2004a). It is, therefore, crucial to characterize both phage and host communities prior to any intended phage applications.

Indeed, phage therapy is complicated by the phenotypic and genotypic complexity of both phage and its bacterial host, consisting of strains with different ranges of susceptibility (Ceyssens et al., 2011; Silva-Valenzuela and Camilli, 2019). In order to make phage therapy viable and wildly shared, it is desirable to find a well-characterized collection of phages that, in combination, cover a broad range of hosts. One proposed solution is a combination of two or more phages with different host ranges in a cocktail to delay the appearance of phage-resistant mutants (Tanji et al., 2004).

In this study, we analyzed the biological features of a collection of 22 clinical *K. pneumoniae* isolates, specifically on the characteristics of multi-locus sequence typing (MLST), plasmid, and antibiotic susceptibility patterns. Using these *K. pneumoniae* isolates, we isolated and characterized three phages native to the hospital sewage. Details of phage host range, genome, morphological characters, and phage-host interactions were established to assess the application of phage therapy against these carbapenem-resistant isolates. Together, our study provides important information regarding phage therapy as an environmentally friendly practice to treat and prevent this important human pathogen.

## Materials and Methods

### Bacterial Strains and Growth Conditions

Twenty-two nonduplicate bacterial isolates of *K. pneumoniae* were collected from Zhongshan Hospital (Shanghai, China) and identified with MALDI-TOF Mass Spectrometry (Bruker, USA) from June 2019 - May 2020. Isolates were routinely grown in either lysogeny broth (LB) containing per liter: tryptophan 10 g, NaCl 10 g, and Yeast extract 5 g with aeration, or, on LB agar (15 g agar per liter) plates at 37°C. The beta-lactamase gene (*bla*_KPC_) was detected by PCR Master Mix (Thermo, CA, USA) with forward primer 5’-TGTAAGTTACCGCGCTGAGG-3’ and reverse primer 5’-CCAGACGACGGCATAGTCAT-3’. All strains were stored at -80°C in LB broth with 25% glycerol (vol/vol) and sub-cultivated before each use. *Klebsiella pneumoniae* strain Kp36, *E. coli* DH5α, and *Salmonella enterica* S7 were used as reference strains, and phage 117 was included as well, which belongs to the *Caudovirales* order, *Podoviridae* family (Tan et al., 2020).

### Multi-Locus Sequence Typing (MLST) and Antibiotic Susceptibility of Bacterial Isolates

MLST with 7 housekeeping genes (*rpoB*, *gapA*, *mdh*, *pgi*, *phoE*, *infB*, and *tonB*) were performed to characterize these 22 isolates, according to the protocol available on the MLST Pasteur website (https://bigsdb.pasteur.fr/klebsiella/klebsiella.html) (Diancourt et al., 2005). The purified MLST amplicons were sent for Sanger sequencing, followed by comparison with those available on the MLST database to determine the sequence type (ST). The VITEK2 compact automated system (bioMérieux, Lyon, France) was used to determine antibiotic susceptibility profiles according to the manufacturer’s instructions. The susceptibility breakpoints were interpreted with reference to the latest documents from the Clinical and Laboratory Standards Institute (CLSI).

### S1-Pulsed Field Gel Electrophoresis (S1-PFGE)

*Klebsiella* with plasmid-mediated carbapenem resistance poses significant challenges in clinical practice. To characterize the plasmid content in each strain, S1 nuclease digestion analyzed with PFGE was performed using a CHEF Mapper XA apparatus (Bio-Rad Laboratories, Hercules, CA). Briefly, the adjusted bacterial suspension mixed in a 1% agarose gel was digested with a proteinase K solution for 2 h at 54°C, then further treated with S1 nuclease. The electrophoresis conditions used in this study were as follows: initial switch time, 3 s; a final switch time, 36 s; gradient, 6 V/cm; angle, 120; temperature, 14°C; and time, 18.5 h. After the electrophoresis run was complete, the gel was stained with 1 mg mL^-1^ ethidium bromide and visualized with the ChemiDoc XRS system (Bio-Rad Laboratories, CA, USA). Genetic similarities were measured using the Dice coefficient and the clustering was carried out by the unweighted-pair group method with arithmetic averages (UPGMA) in BioNumerics (Applied Maths, St-Martens-Latern, Belgium).

### Isolation and Proliferation of Phage

Phages were isolated using an enrichment protocol as described previously (Tan et al., 2014). Briefly, infiltrated hospital sewage was mixed with the same volume of 2 × LB broth and incubated at 37°C for 6 h to stimulate proliferation of total abundances of specific phages targeting the *K. pneumoniae* isolates above. Aliquots of enrichment samples were centrifuged (12,000×*g*, 10 min, 4°C) and sterilized with 0.1% chloroform. The presence of *K. pneumoniae*-phages in the hospital sewage samples was detected by double-layer agar assays with different fold dilutions of the cell-free spent supernatant spent from the enrichment assay. In order to obtain individual phages, single plaques were picked and purified three times with their corresponding hosts. Phage stocks were enriched and titrated using double-layer agar assays as described previously and stored at 4°C before use (Kropinski et al., 2009).

### Phage-Host Range Assay

The host ranges of obtained phages and phage 117 were determined against 22 clinical isolates along with a well-characterized reference *K. pneumoniae* strain Kp36, *E. coli* DH5α, and *Salmonella enterica* S7 by standard spot assays (Tan et al., 2020). Aliquots of 2 µL diluted phage lysate (~10^5^ PFU ml^-1^) were spotted on lawns containing 4 mL top agar mixed with 300 µL mid-log phase bacteria. After incubating at 37°C for 12 h, the spots were assessed by the turbidity of plaques and characterized as strong infection (clear), weak infection (turbid), or no inhibition (no plaque). The experiment was undertaken as independent duplicates.

### Susceptibility of Strain ZS15 to Phage Infection and Isolation of Phage-Resistant Mutants

Overnight bacterial cultures were 1,000-fold diluted into LB broth and grown to an exponential phase with an OD600 at 0.3 and infected with single phages or phage cocktail (NL_ZS_1, NL_ZS_2, and NL_ZS_3) at a multiplicity of infection (MOI) of 0.01. The suspension was incubated at 37°C with aeration and samples were harvested at 1-h intervals. The phage lysis efficiency was determined by optical density measurements at OD600_nm_. The experiment was done in triplicate along with parallel control cultures without phages. At time point 8-h, single phage lysates were streaked onto LB agar plates and individual colonies were subsequentially purified to determine phage susceptibility by spot test assays as described above.

### Phage DNA Extraction and Genomic Analysis

For the extraction of phage DNA, 2 mL aliquots of phage lysate with a titer of approximately 10^9^ PFU mL^-1^ was digested with DNase I (Thermo, CA, USA) and RNase A (Promega, USA) to remove any residual bacterial genomic DNA and RNA according to the manufacturer’s protocol. After digesting for 1.5 h at 37°C, EDTA (Thermo, CA, USA) was added to a final concentration of 20 mM to inactivate DNase I and RNase A. Total phage DNA was extracted using the Qiagen DNeasy Blood and Tissue kit (Qiagen, CA, USA) following the manufacturer’s instructions. Genomic DNA was sent to Sangon Biotech (Shanghai, China) and sequenced using an Illumina HiSeq sequencer.

The raw sequence containing the phage genome was quality controlled and trimmed with FastQC 0.11.2 (https://www.bioinformatics.babraham.ac.uk/projects/fastqc/) and Trimmomatic 0.36 (http://www.usadellab.org/cms/?page=trimmomatic). The trimmed reads were assembled into a single raw contig *via* SPAdes 3.5.0. (http://cab.spbu.ru/software/spades/). Genes were predicted and annotated using Rapid Annotation using Subsystem Technology (RAST, http://rast.nmpdr.org/). Phage virulence factor was examined using Virulence Factor Database (VFDB, http://www.mgc.ac.cn/VFs/). Phylogenetic relationship between the genome of phage NL_ZS_1, NL_ZS_2, and NL_ZS_3 and the genomes of related phages were analyzed using CLC Main Workbench (CLC, Qiagen). To construct the phylogenetic tree of phage terminase large subunits, the reference amino acid sequences of were collected from the NCBI database. The terminase large subunits were aligned, and neighbor-joining trees were created using Jukes-Cantor model with a bootstrap analysis of 1000 replicates.

### Bacteriophage Morphology by Transmission Electron Microscopy (TEM)

Based on the genomic differences, phages were selected for examination by TEM. Phage stocks were back-diluted in a SM buffer (50 mM Tris-Cl, pH 7.5, 99 mM NaCl, 8 mM MgSO_4_, 0.01% gelatin) to a titer of 10^8^ PFU mL^-1^. Diluted phage lysates were then placed on the surface of Formvar carbon-coated grids (Sigma-Aldrich, St. Louis, MO, USA) for 5 min to allow adsorption. Grids were stained with 2% sodium phosphotungstate for 2 min prior to being washed in a drop of distilled water and air-dried before being examined using a JEM-2100 microscope operated at 80 kV.

## Results

### Isolation and Characterization of *K. pneumoniae* Strains

The emergence of carbapenem-resistant *K. pneumoniae* (CRKP) has become one major burden of healthcare-associated infection due to the dispersion of bacteria carrying the *bla*_KPC_ gene (Bush and Bradford, 2016). KPC was first found in carbapenem-resistant *K. pneumoniae* strains in 2001 in North America, and now KPC-producing strains can be frequently isolated from nosocomial samples. It is, therefore, essential to determine and characterize the nosocomial spread of KPC clones and their corresponding phenotype and genotype. Together, 22 carbapenem-resistant *K. pneumoniae* strains were recovered from various clinical specimens (Zhongshan Hospital, Shanghai, China). According to the *K. pneumoniae* MLST database, all the 22 clinical isolates belonged to ST11 (allelic profile 3-3-1-1-1-1-4), known as the predominant clone of KPC-producing *K. pneumoniae* in China (**Table 1**). Despite more than 100 different sequence types (STs) being reported worldwide, the mass dissemination of carbapenemase-producing *K. pneumoniae* has been restricted largely to ST258, ST11, ST340, and ST512 (Pitout et al., 2015).

**Table 1:** MLST types and antibiotic susceptibility profiles of the 22 *K. pneumoniae* isolates.

Minimum Inhibitory Concentration (μg/mL)																			
Strain	MLST	*bla*_KPC_	TIM	TZP	CAZ	CPZ	FEP	ATM	IMP	MEM	AMK	TOB	CIP	LVX	DOX	MIN	TGC	CST	SXT
ZS1	ST11	Yes	≥128	≥128	≥64	≥64	≥32	≥64	≥16	≥16	≥64	≥16	≥4	≥8	2	4	≤0.5	≤0.5	≤20
ZS2	ST11	Yes	≥128	≥128	≥64	32	≥32	≥64	≥16	≥16	4	8	≥4	≥8	2	8	1	≥16	≤20
ZS3	ST11	No	≥128	≥128	≥64	≥64	≥32	≥64	1	1	≥64	≥16	≥4	≥8	4	≥16	2	≤0.5	≤20
ZS4	ST11	Yes	≥128	≥128	32	≥64	≥32	≥64	0.5	≤0.25	≥64	≥16	≥4	≥8	2	8	1	≤0.5	≤20
ZS5	ST11	Yes	≥128	≥128	≥64	≥64	≥32	≥64	≥16	≥16	4	≤1	≥4	≥8	8	≥16	4	≤0.5	≤20
ZS6	ST11	Yes	≥128	≥128	≥64	≥64	≥32	≥64	≥16	≥16	≥64	≥16	≥4	≥8	1	2	≤0.5	≤0.5	≤20
ZS7	ST11	Yes	≥128	≥128	≥64	≥64	≥32	≥64	≥16	≥16	4	≤1	≥4	≥8	≥16	≥16	≥8	≤0.5	≤20
ZS8	ST11	Yes	≥128	≥128	≥64	≥64	≥32	≥64	≥16	≥16	≥64	≥16	≥4	≥8	4	8	2	≤0.5	≤20
ZS9	ST11	Yes	≥128	≥128	≥64	≥64	≥32	≥64	≥16	≥16	≥64	≥16	≥4	≥8	4	8	2	≤0.5	≤20
ZS10	ST11	Yes	≥128	≥128	≥64	≥64	≥32	≥64	≥16	≥16	≥64	≥16	≥4	≥8	≥16	≥16	4	4	≥320
ZS11	ST11	Yes	≥128	≥128	≥64	≥64	≥32	≥64	≥16	≥16	≥64	≥16	≥4	≥8	2	8	2	≤0.5	≤20
ZS12	ST11	Yes	≥128	≥128	≥64	≥64	≥32	≥64	≥16	≥16	≥64	≥16	≥4	≥8	2	8	1	≤0.5	≤20
ZS13	ST11	Yes	≥128	≥128	≥64	≥64	≥32	≥64	≥16	≥16	≥64	≥16	≥4	≥8	2	8	≤0.5	≤0.5	≤20
ZS14	ST11	No	≥128	≥128	32	≥64	≥32	≥64	1	0.5	≥64	≥16	≥4	≥8	2	8	≤0.5	≤0.5	≤20
ZS15	ST11	Yes	≥128	≥128	≥64	≥64	≥32	≥64	≥16	≥16	≥64	≥16	≥4	≥8	2	8	8	≤0.5	≤20
ZS16	ST11	Yes	≥128	≥128	≥64	≥64	≥32	≥64	≥16	≥16	≥64	≥16	≥4	≥8	4	≥16	2	≥16	≤20
ZS17	ST11	Yes	≥128	≥128	≥64	≥64	≥32	≥64	≥16	≥16	≥64	≥16	≥4	≥8	4	8	1	≤0.5	≤20
ZS18	ST11	Yes	≥128	≥128	≥64	32	≥32	≥64	≥16	8	8	≤1	≥4	≥8	≤0.5	2	≤0.5	≤0.5	≤20
ZS19	ST11	No	≥128	≥128	8	≥64	≥32	≥64	≤0.25	≤0.25	≥64	≥16	≥4	≥8	2	4	≤0.5	≤0.5	≤20
ZS20	ST11	Yes	≥128	≥128	≥64	≥64	≥32	≥64	≥16	≥16	≥64	≥16	≥4	≥8	1	4	≤0.5	≤0.5	≤20
ZS21	ST11	Yes	≥128	≥128	≥64	≥64	≥32	≥64	≥16	≥16	≥64	≥16	≥4	≥8	1	4	≤0.5	≤0.5	≤20
ZS22	ST11	Yes	≥128	≥128	≥64	≥64	≥32	≥64	≥16	≥16	≥64	≥16	≥4	≥8	1	2	≤0.5	≤0.5	≤20

Antibiotic abbreviations: TIM, ticarcillin/clavulanic acid; TZP, piperacillin/tazobactam; CAZ, ceftazidime; CPZ, cefoperazone; FEP, cefepime; ATM, aztreonam; IPM, imipenem; MEM, meropenem; AMK, amikacin; TOB, tobramycin; CIP, ciprofloxacin; LVX, levofloxacin; DOX, doxycycline; MIN, minocycline; TGC, tigecycline; CST, colistin; SXT, Trimethoprim/Sulfamethoxazole.

### ß-Lactamase Content and Plasmid Characterization

In addition, the *bla*_KPC_ gene was detected in 19 out of 22 isolates, indicating the prevalence of β-lactamase nosocomial pathogens (**Table 1**). The *bla*_KPC_ gene can be disseminated between species by horizontal (lateral) gene transfer (HGT) *via* various means, such as plasmids, transposons, prophages, and integrative conjugative elements (ICEs), playing an important effect in generating genotypic heterogeneity and phenotypic variation among bacterial populations (Hardiman et al., 2016; Botelho and Schulenburg, 2020). For instance, transfer of interspecies KPC can occur through the dissemination of mobile genetic elements between strains of *S. aureus* (Goren et al., 2010 **).**

**Figure 1:**
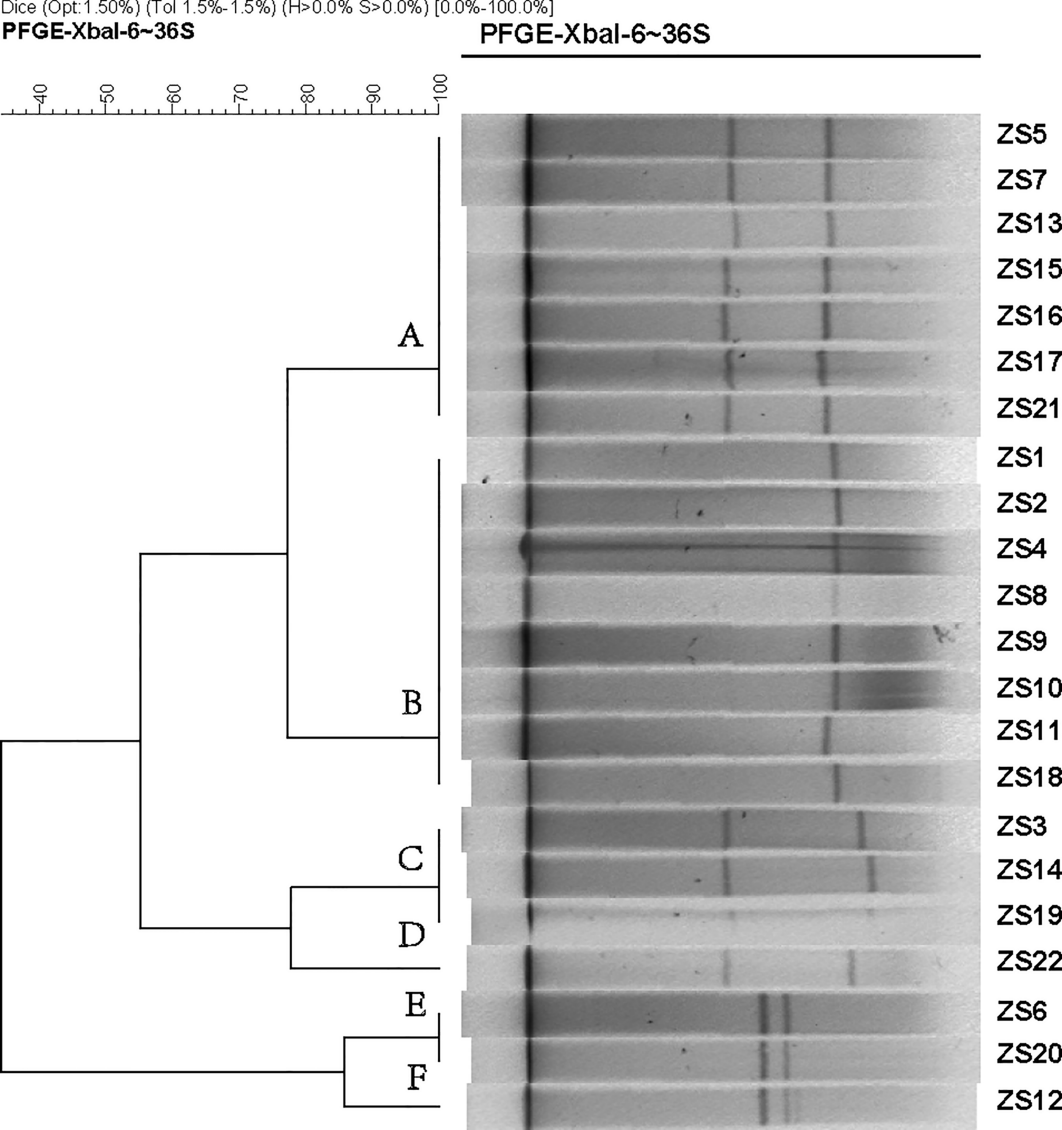
Dendrogram generated by BioNumerics software (Version7.1, Applied Maths, St-Martens-Latem, Belgium) showing cluster results of molecular epidemiology S1-PFGE analysis of 22 *K. pneumoniae* isolates. Band-based similarity coefficients were calculated using Dice coefficient and unweighted pair group average method algorithm (UPGMA). *Salmonella enterica* serotype Braenderup strain H9812 was digested with XbaI and used as a molecular size marker. The electrophoresis conditions were as follows: initial switch time, 3 s; a final switch time, 36 s; gradient, 6 V/cm; angle, 120; temperature, 14°C and time, 18.5 h.

Since pathogenicity and antimicrobial resistance genes are mainly coded by conjugative plasmids and integrative conjugative elements (ICEs, also known as conjugative transposons), in order to identify the subtypes of *K. pneumoniae* ST11, pulsed-field gel electrophoresis with S1 nuclease (S1-PFGE) were utilized to resolve the prevalence of plasmid fingerprints of all the 22 isolates, particularly large ones. All groups harbored one to two plasmids ranging from 20 Kbp and 250 Kbp, which were clustered into 6 primary clades (A, B, C, D, E, and F) (**Figure 1**). Clade A and B were the most common types, while the other clades (C, D, E, and F) only accounted for 31.8%. Thus, it appears that S1-PFGE is a highly discriminative molecular typing method capable of effectively discriminating ST11 clones into several different subtypes, indicating much heterogeneity among these KPC-producing isolates.

### Preliminary Antibiotic Susceptibility Testing of *K. pneumoniae* Isolate

Individual antimicrobial data can be found in **Table 1**. For ticarcillin/clavulanic acid, piperacillin/tazobactam, cefepime, aztreonam, ciprofloxacin, and levofloxacin, drug resistance was detected in all strains. However, low percentages of resistance to doxycycline, minocycline, tigecycline, colistin, and trimethoprim/sulfamethoxazole were also observed for most strains. Of these, 17 out of the 22 isolates become resistant to both imipenem and meropenem with a MIC of ≥16 µg mL^-1^, while the other 5 were both sensitive to imipenem and meropenem, except for strain ZS18 which was resistant to imipenem but intermediate to meropenem. For these 5 sensitive isolates, 2 of them were confirmed by PCR and sequencing to have the *bla*_KPC_ gene. Interestingly, strain ZS4 harboring *bla*_KPC_ was susceptible to imipenem and meropenem, perhaps indicating the resistance is associated with changes in gene expression at the mRNA levels (Senchyna et al., 2019).

### Isolation and Characterization of Phages

**Figure 2:**
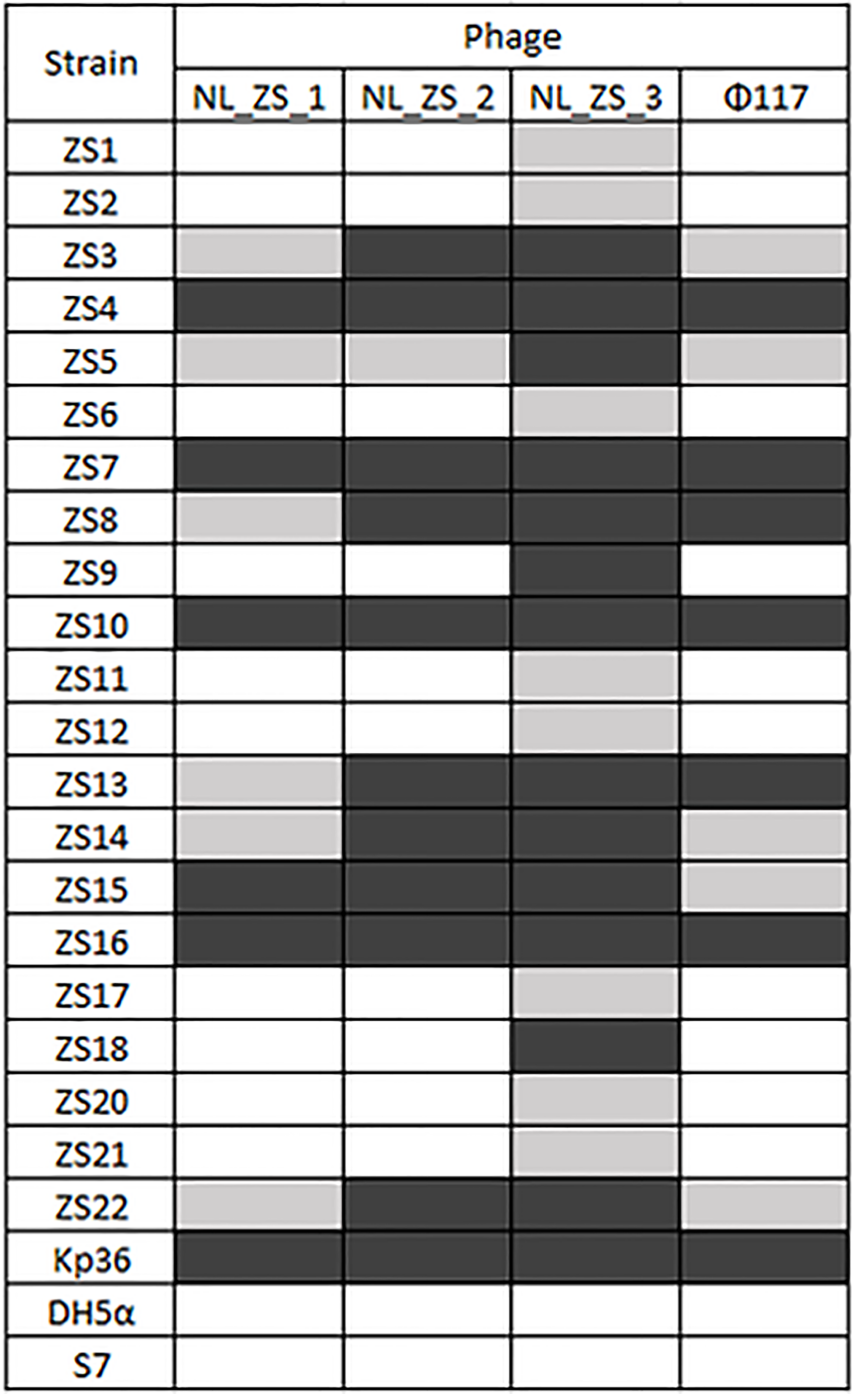
Phage-host range assay of the 4 phages (columns) against the 22 K. *pneumoniae* strains and the reference strain Kp36, DH5α, and S7 (rows). *Klebsiella pneumoniae* phages displayed broad and narrow host ranges. Black, grey and white indicate infection, weak infection and no plaque formation, respectively.

Three phages (NL_ZS_1, NL_ZS_2, and NL_ZS_3) were isolated from the hospital sewage using the enrichment protocol. The host range of the phages (NL_ZS_1, NL_ZS_2, NL_ZS_3, and phage 117) and their lytic efficiency against all the 23 *Klebsiella* isolates and two *Enterobacterales* strains (*E. coli* DH5α, and *S. enterica* S7) was assessed using the spot test assay. According to the host range profile, the phages were able to infect all the 23 *K. pneumoniae* bacterial isolates, but none of the phages were able to produce plaques on the strain *E. coli* DH5α or *S. enterica* S7 (**Figure 2**), indicating all the phages used in this study failed to lysis relevant *Enterobacterales* species. The host range patterns and lytic efficiency showed two unique profiles. Based on these profiles, phages were divided into two major groups. Specifically, phage NL_ZS_3 displayed the broadest host range, while phage NL_ZS_1, phage NL_ZS_2, and phage 117 showed a relatively narrow host range, infecting only ~50% of the tested *K. pneumoniae* strains (**Figure 2**). The host range differences between the phages observed in this study are likely due to the different phage characterization, i.e., efficiency of adsorption and lytic efficiency in its potential hosts.

### Morphology and Genome Sequence of Phages

**Figure 3:**
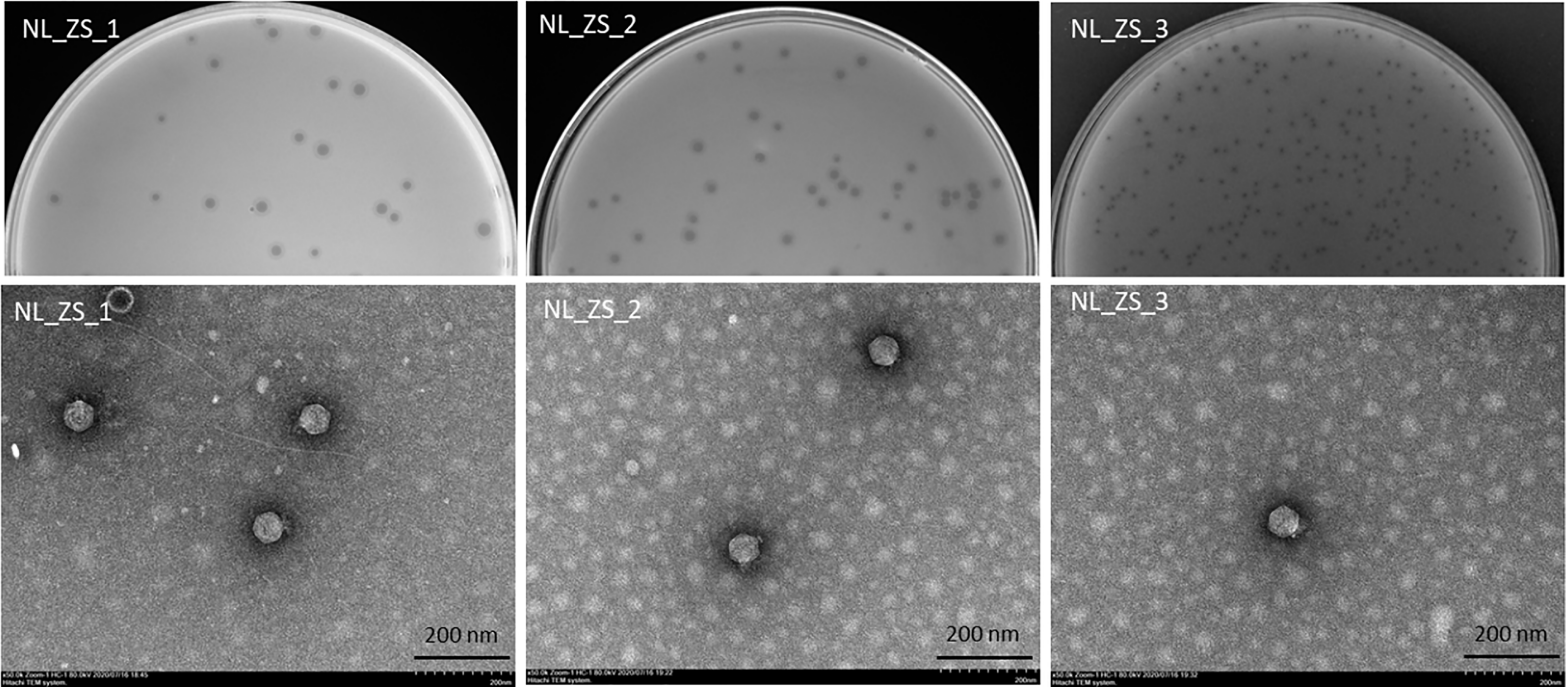
Plaque images (up) and their corresponding TEM micrographs (below). Phages were stained with 2% sodium phosphotungstate and were found to have short small podophage morphology. Scale bar, 200 nm.

All phage lysates formed transparent plaques on strain ZS15, which are considered as an indicator of strong lysis. Plaque morphologies of all phages presented a zone of clear lysis on the host strain ZS15, while a halo zone was detected on the plaques of both phages NL_ZS_1 and NL_ZS_2, indicating the production of polysaccharide depolymerase (**Figure 3**). Depolymerase activity is usually encoded by their tail fibers or tail spikes protein on the baseplate. Since depolymerases target and destroy cell wall structures, they have been suggested as potential antimicrobial agents to conventional antibiotics.

**Figure 4:**
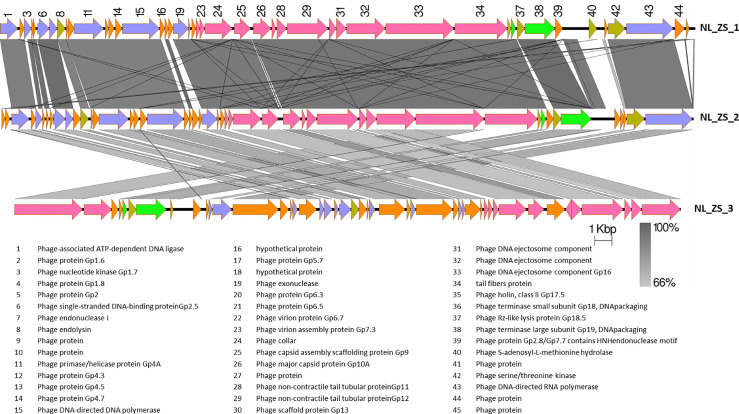
The comparison of the whole genome sequences among three *K. pneumoniae* phages (NL_ZS_1, NL_ZS_2, and NL_ZS_3) with Easyfig using the BLASTN algorithm. Grey lines connect regions indicates nucleotide identity >66%, and are gradient-colored according to their similarity; darker shade of grey represents higher identify. The designation of the open reading frames of NL_ZS_1 is shown on the top. Conserved ORFs are color-coded with blue arrows representing the DNA replication module, yellow arrows representing host cell lysis module, pink reparenting the capsid and tail morphogenesis module, green arrows representing the DNA packaging module, and borrow arrows represent genes with no known function.

**Figure 5:**
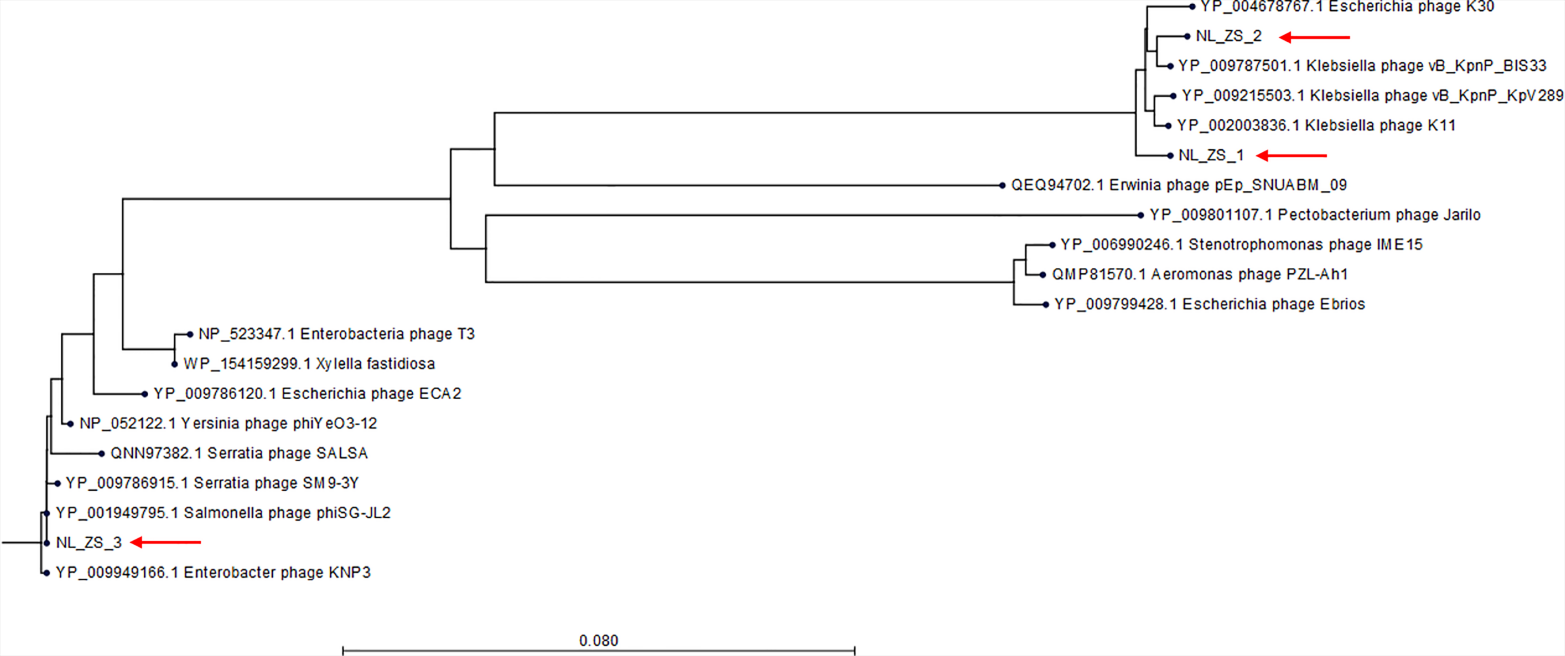
Phylogenetic tree was constructed based on the amino acid sequence of the larger terminase subunit of phages NL_ZS_1, NL_ZS_2, and NL_ZS_3 and phages sharing homology sequence identity retrieved from GenBank (https://www.ncbi.nlm.nih.gov/). The evolutionary history of 20 larger terminase subunit amino acid sequences were aligned and inferred using Neighbor Joining method and 1000 bootstrap replicates (CLC Genomics Workbench 8).

Based on TEM analysis, all the phages belong to the family *Podoviridae* of *Caudovirales* order, having short, non-contractile tails (~11 nm in length) and icosahedral heads (~61 nm diameter) (**Figure 3** and **Table 2**). In addition, total genomic DNA was extracted from each of the three phages, and their genomes were sequenced by Illumina MiSeq. The assembly sizes of these three phages ranged from 38 kb to 40 kb, having a G+C content ranging between 50% to 53%, with 45 to 49 predicted protein-coding genes (**Table 2**). All phage genomes (GenBank accession: phage NL_ZS_1, MT813140; phage NL_ZS_2, MT813141; and phage NL_ZS_3, MT813142) were submitted to RAST (Rapid Annotation using Subsystem Technology) and further annotated by the available pipeline. No identifiable virulence genes, antibiotic resistance genes, or genes involved in lysogenic cycles were detected in the genomes. Therefore, it appears that those phages isolated in this study can satisfy the safety assessment based on their complete genome sequences. Progressive multiple genome alignments were analyzed using Easyfig software (https://mjsull.github.io/Easyfig/) (**Figure 4**) to determine the relatedness of three *K. pneumoniae* phages. A considerable relation was shown between phage NL_ZS_1 and phage NL_ZS_2, while phage NL_ZS_2 displays a relatively low homologous region with phage NL_ZS_3. Likely, phylogenetic analysis of the large subunit of the terminase of phage NL_ZS_1, NL_ZS_2, and NL_ZS_3 reveals their close relation to large terminase subunits of other phages, specifically, phage NL_ZS_1 and phage NL_ZS_2 display high similarities to *Klebsiella* phage K11, BIS33, and KpV289, while phage NL_ZS_3 was closely related to *Salmonella* phage phiSG-JL2 (**Figure 5**). Overall, these results showed high relation to the phage-host range pattern, demonstrating phage susceptibility correlates with phage core genome, such as genes involved in the phage tail fibers.

**Table 2:** Morphological and genomic characterization of phages isolated from the local hospital sewage.

Phage	Family	Host	Head diam(nm)	Head length(nm)	Tail diam(nm)	Tail length(nm)	Genome size (bp)	GC%	Genome type	Predicted genes	Source
NL_ZS_1	Podoviridae	ZS15	63	58	13	14	40,428	53	dsDNA	45	Sewage
NL_ZS_2	Podoviridae	ZS15	58	63	13	9	40,222	52.8	dsDNA	49	Sewage
NL_ZS_3	Podoviridae	ZS15	63	63	13	11	38,741	50.7	dsDNA	46	Sewage

### Phage Growth Characteristics in LB Broth

**Figure 6:**
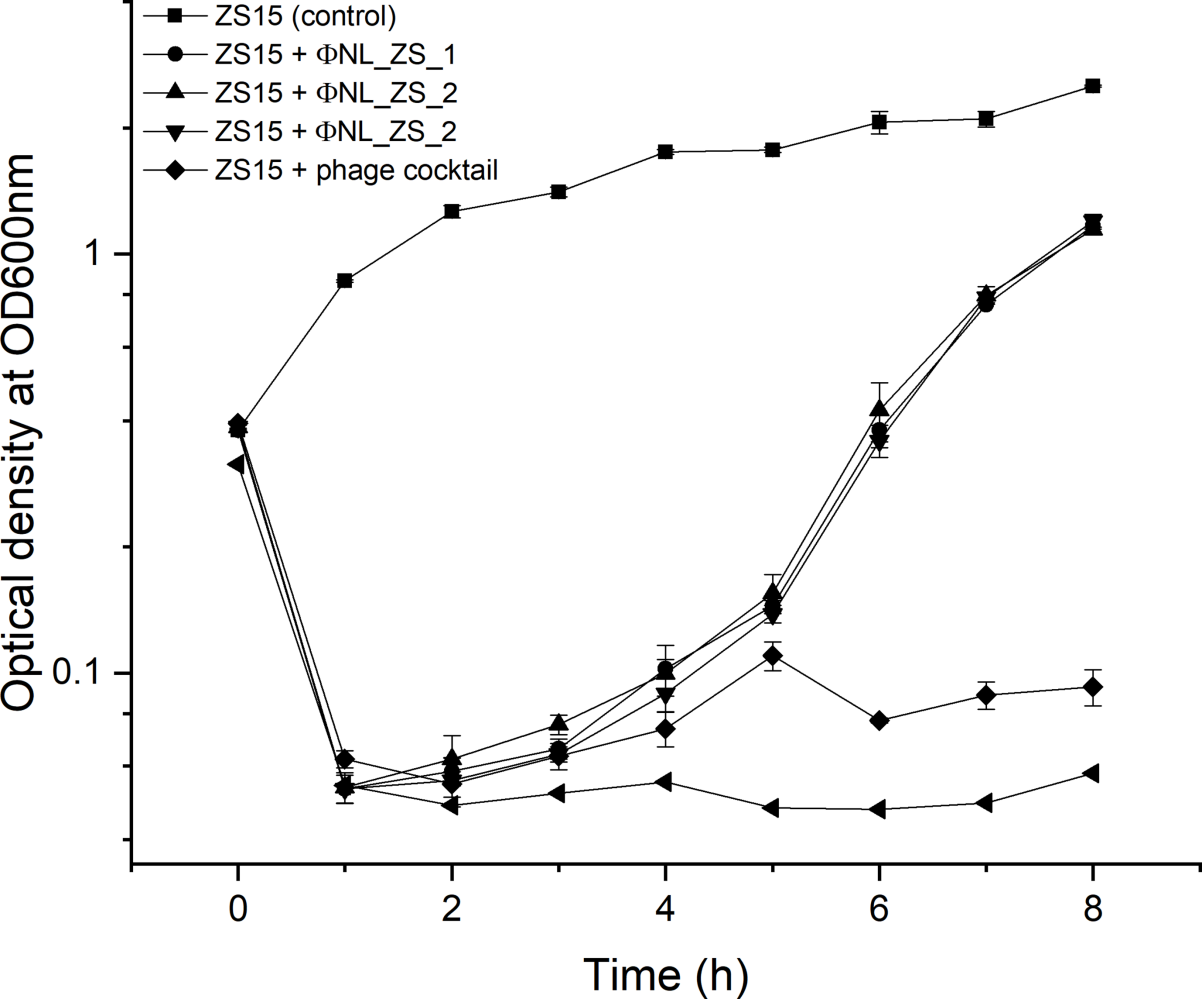
Inhibition assay of single phages and phage cocktail (NL_ZS_1, NL_ZS_2, and NL_ZS_3) in the treatment at a MOI of 0.1 and control without phage in cultures of *K. pneumoniae* ZS15. Optical density (OD600) was measured at 1-h intervals over an 8-h incubation period. Error bars represent the standard deviations from all experiments, carried out in triplicate.

To examine the efficiency of phage infection, *K. pneumoniae* ZS15 was infected in liquid cultures with single phages and phage cocktail (NL_ZS_1, NL_ZS_2, and NL_ZS_3) at a MOI of ~0.01, respectively, and their inhibition was assessed by bacterial growth following the measurement of the optical density of the bacterial cultures with and without phages. An inhibitory effect of phage NL_ZS_1 and phage NL_ZS_2 was observed in the *in vitro* inhibition assay with a drastic decline in optical density on the initial control of the host population for 1.5 h followed by regrowth of the resistant population (**Figure 6**). Likely, a similar trend in the phage-amended cultures of ZS15+ NL_ZS_3 was observed in the first 5 h, however, it was followed by a less pronounced decrease and finally stabilized after 6 h (**Figure 6**). To assess the applicability of the phage cocktail, we performed the same growth curve inhibition analysis on the strain ZS15. As expected, the phage cocktail showed a stronger distribution of growth inhibition than that of phage NL_ZS_3, indicating the strain ZS15 can be suppressed for longer by the phage cocktail combining three different phages. After 8 h, the OD600 nm of the phage-amended cultures of group 1 (ZS15+phage NL_ZS_1 and ZS15+phage NL_ZS_2), group 2 (ZS15+phage NL_ZS_3), and group 3 (phage cocktail) stabilized at 1.95, 1.96, 0.09, and 0.05, corresponding to 52% to 98% of the final OD in the control group, respectively (**Figure 6**). Together, these results demonstrate that phage cocktail were more effective in reducing bacterial densities compared with the single phage, thus prolonging the usable time of the phage cocktail, and increasing the efficiency in phage application.

Despite *K. pneumoniae* ZS15 displaying a strong susceptibility to infection by all three phages, phage NL_ZS_3 was better at suppressing resistance to host strain ZS15 over phage NL_ZS_1 and NL_ZS_2. Bacteria are known to rapidly develop phage resistance when subjected to predation by lytic phages in a laboratory medium (Labrie et al., 2010). By screening phage resistance mutants with spot test, we attempted to better understand the inhibition differences among each of the phage-amended cultures. To do this, phage insensitive mutants were isolated from phage lysate cultures during the incubation for the analysis of changes in their susceptibility upon phage selection. Based on the results, we observed that phage-amended cultures (ZS15+phage NL_ZS_1, ZS15+phage NL_ZS_2, and ZS15+phage NL_ZS_3) were dominated by resistant non-mucoid clones after 8 h of incubation, indicating conversion of mucoid *K. pneumoniae* strain ZS15 to non-mucoid variants by consequence of selective pressure operated by phages (**Table 3**). However, in the ZS15+phage NL_ZS_3 cultures, heterogeneity in phage susceptibility was observed (**Table 3**). Out of 30 K*. pneumoniae* ZS15 isolates, 2 (~6.7%) were fully sensitive to phage NL_ZS_3 and displayed the hypermucoviscosity (HV) phenotype as the parental strain ZS15, and partially sensitive (i.e., reduced phage susceptibly when compared to the parental strain) subpopulations (~50%) were isolated as well and did not revert back to the wild-type phenotype despite three rounds of purification. Interestingly, 43.3% were fully resistant to phage NL_ZS_3, however, a small fraction (~30%) of phage NL_ZS_3 resistant mutants reverted back to the parental phenotype after three rounds of purification, suggesting different anti-phage mechanisms were allowing coexistence of phage sensitive mutants and that their lytic phages could exist between bacterial host ZS15 and phage NL_ZS_3. We do not know, however, the dynamics of phenotype heterogeneity over time or how long such persistence can last before heritage mutations dominate the entire population, since phage susceptibility was only measured at 8 h,

**Table 3:** Phage susceptibility properties of *K. pneumoniae* ZS15 isolates obtained from cultures enriched with the phages.

Percentage of phage susceptibility properties of *K. pneumoniae* ZS15 isolates (%)			
Phage	Resistant	Partially sensitive	Sensitive
NL_ZS_1	100%	0	0
NL_ZS_2	100%	0	0
NL_ZS_3	43.3%	50%	6.7%

“Resistant” indicates that isolates were not susceptible to phage infection in the spot test. “Partially sensitive” indicates that isolates had a reduced susceptibility to the phage. “Sensitive” indicates that isolates were fully sensitive to the phage.

### Anti-Phage Defense-Mediated Altered Sensitivity to Tigecycline

Previous studies have shown that evolutionary phage-host interactions affect phage resistance and antibiotic susceptibility both positively and negatively, and are highly genotype-dependent in bacterial populations (Comeau et al., 2007; Chan et al., 2016; Burmeister et al., 2020). We started to question whether phage-resistant mutants changed their antibiotic susceptibilities *via* antagonistic coevolution between hosts and parasites in LB broth. To investigate this further, we performed Vitek 2 assays for antimicrobial susceptibility, testing phage NL_ZS_3-resistant mutants (ZS15- NL_ZS_3R) and their parental phage susceptible strains (ZS15). Interestingly, for tigecycline susceptibility testing of carbapenem-resistant *K. pneumoniae* ZS15 strain, we noticed that strain ZS15 evolved resistance to phage NL_ZS_3, while simultaneously becoming sensitive to tigecycline with the minimum inhibitory concentration shifting from 8 µg mL^-1^ to 2 µg mL^-1^. Unfortunately, we were unable to identify potential genes related to phage selection and antibiotic sensitivity during the interactions between strain ZS15 and phage NL_ZS_3. Overall, these results indicate that an evolutionary trade-off between phage resistance and antibiotic resistance. Further, comparative genomic sequence analyses are needed to elucidate the underlying mechanisms by which restoration of antibiotic susceptibility occurs.

## Discussion

*Klebsiella pneumoniae* is a common nosocomial pathogen that causes difficult-to-treat infections worldwide. The prevalence and distribution of *bla_KPC_* gene in *K. pneumoniae* is increasing, making carbapenem resistance isolates difficult to treat due to the lack of effective and safe alternative options. Successful application of phage therapy in treating *K. pneumoniae* caused infections requires a detailed understanding of the phenotypic and genotypic diversity of *K. pneumoniae* pathogens and phages. In this study, we characterized 22 carbapenem-resistant *K. pneumoniae* strains and 3 lytic phages. Further, *K. pneumoniae* strain ZS15 was selected for more detailed studies of phage lytic potential and phage-host interactions.

### Diversity of *K. pneumoniae* ST11 Isolates

The clonal relationship between *K. pneumoniae* strains examined in the present study belonged to ST11 irrespective of time or place of isolation. ST11 is among the most important clinical dominant in Asia and South America and has been reported in different regions worldwide since its emergence (Qi et al., 2011). In 2018, a fatal outbreak of ST11 carbapenem-resistant hypervirulent *K. pneumoniae* in a Chinese hospital has caused high mortality and morbidity (Gu et al., 2018; Yao et al., 2018). Likewise, ST258, single-locus variants of ST11 (the *tonB* allele distinguishes the two sequence types), has emerged as an important cause of hospital death in the United States and other countries, and is largely responsible for the global spread of KPC (Toth et al., 2010). However, the molecular linage between ST11 and ST258 largely remains unknown. Recent comparative genomic analyses showed ST258 is a hybrid with ~80% of the chromosome homology to ST11 strains, while the remaining 20% display high similarity to the ST442 isolates (Chen et al., 2014). Despite MLST does not provide sufficient discrimination for the 22 pathogenic isolates or resolve differences across spatial and temporal sac ales, understanding the molecular evolutionary history of bacterial isolates is an important step towards the development of diagnostic and potential therapeutic strategies to combat infections caused by multidrug-resistant *K. pneumoniae*.

The antibiotic susceptibility profile presents a worrying trend of antimicrobial resistance, as carbapenem resistance was seen among all *Klebsiella* isolates. Treatment options for infections with carbapenem non-susceptible *K. pneumoniae* are very limited, leaving colistin as the last treatment option to fight against these infections. Out of 22 strains, 2 isolates (strain ZS2 and ZS16) display resistance to colistin. Currently, there is little understanding of molecular mechanisms underlying antibiotic resistance and its impact on virulence remains unclear, despite ~86% of strains being confirmed to be carbapenemase gene positive. A recent sequence of complete *K. pneumoniae* genomes showed that genes coding for resistance to antibiotics or virulence factors is located in the plasmid (David et al., 2020). Moreover, the spread of these resistant plasmids into the normal gut flora poses an even great public health threat. To recapitulate the contents and diversity of the plasmids of genetically related *K. pneumoniae* isolates, it is important to track the spread of its plasmids between each isolate. In this study, S1-PFGE showed four clusters of the CRKp producing strains that corresponded to ST11, representing a high level of genetic diversity. Together, these results provide important insights into relatively narrow phylogenetic and phenotypic groups of bacteria, emphasizing the complexity of the development of alternatives to treat this pathogen. Future work will seek to understand the dissemination of these plasmids and their potentials of converting any *K. pneumoniae* strains with no existing antimicrobial resistance or virulence determinants into CRKp strain.

### Phage Isolation and Diversity

Generally, phages are widespread and genetically diverse in almost all environments, especially in sewage, known as a fertility source for many pathogens. The high occurrence of *K. pneumoniae* phages in the collected samples (~72%) suggested the *K. pneumoniae* phages are widespread in the local hospital sewage. The phages displayed two distinguished host ranges when tested against the strain collection. Only 1 morphological family, *Podoviridae*, was represented, and the phages ranged in genome size from 38 kb to 40 kb. Due to the limited sample numbers, we do not know to what extent these isolated phages represent the true diversity and structure of local hospital sewage phage communities. Nevertheless, it would be interesting to experimentally study the viral metagenomics that is not dependent on cell culture approaches to expand our understanding of phage genomic diversity from the evolutionary perspective and perhaps genetic exchanges in response to the selection pressure from the bacterial host.

Indeed, each phage has its own particular host range, defined as the set of strains permissive for its infection. Some broad host range phages can infect multiple species of bacteria. For instance, phage KVP40 is able to infect *Vibrio anguillarum*, *V. parahaemolyticus*, *V. harveyi*, and *V. cholerae* (Matsuzaki et al., 1992); bacteriophage Mu infects several species, including *E. coli*, *Citrobacter freundii*, *Shigella sonnei*, *Enterobacter*, and *Erwinia* (Ross et al., 2016). However, host ranges can be limited by diverse phage receptors on the surface of bacteria. In this study, phage NL_ZS_3 displayed a relatively broad host range, while phage NL_ZS_1, phage NL_ZS_2, phage 117 shared identical relatively narrow host ranges, suggesting that they might use similar phage binding receptors to recognize bacterial phage receptors (olonic acid) in their susceptible hosts. This is interesting as bioinformatic analysis showed that phage-binding receptors of phage NL_ZS_1 and phage NL_ZS_2 were identical based on the amino acid sequence. The possible explanation for host range difference could be phage NL_ZS_3 used a conservative apparatus as its phage receptors, such as outer membrane proteins, lipopolysaccharides, flagella, or pili. Moreover, several copies of genes encoding phage tails or tail fibers were present in the phage NL_ZS_3, which opens the possibility that phage NL_ZS_3 may use a primary receptor to initiate attachment but require a secondary cell surface receptor to irreversibly bind to the bacterial host for injection, permitting more flexibility to develop on a wider range of hosts. For example, the T4 phage uses its long tail fibers to recognize lipopolysaccharide and the outer membrane protein C, present on the bacterial surface, and this reversible attachment allows the phage to move along the surface and find a suitable site to inject its DNA (Tétart et al., 1996; Baslé et al., 2006).

### Phage-Host Interactions in Strain ZS15

Isolation and characterization of bacterial isolates during the LB broth experiments confirmed the rapid development of phage resistance and subsequently dominated the bacterial populations in the phage NL_ZS_1- and phage NL_ZS_2-amend cultures, which is regarded as a significant barrier to the successful application of phage therapy (Koskella and Meaden, 2013). Interestingly, in the current experiment with strain ZS15, phage NL_ZS_ 3 exposures led to the rapid development of phenotypic diversity (i.e., resistant, partially sensitive, or resistant) within a population that enables survival of fluctuating, suggesting different anti-phage mechanisms were allowing coexistence of phage sensitive mutants and their lytic phages. Since phage susceptibility was only measured at 8 h, we do not know the dynamics of phenotype heterogeneity over time or how long such persistence can last before heritage mutations dominate the entire population. It is, therefore, crucial to characterize the phage-host interaction regarding the phenotypic and genetic diversification in bacteria and phages prior to any intended phage applications.

A common form of developing phage resistance for bacterial hosts is associated with loss or altered structure of the receptors to block the potential phage adsorption in order to avoid infection (Labrie et al., 2010). Such changes are directly linked to the key step in recognition between phage receptor-binding proteins (RBP) located on tail fibers, and phage receptors encoded on the susceptible bacterial host cell surface (Bertozzi Silva et al., 2016). In general, the fitness costs resulting from receptor mutation (i.e., motility, nutrient transport, or antimicrobial susceptibility) make the mutants unable to compete with phage-susceptible strains in both environmental samples and infection models, even in the presence of phage predation (Seed et al., 2012; Sumrall et al., 2019). Understanding the evolutionary implication of phage-receptor interactions will be beneficial for predicting how phages interact with their host, as phage receptor mutation-mediated phenotypic variabilities may result in exopolysaccharide associated fitness cost. Such characterization of phage resistance evolution will be used as a guide for selecting proper phage targeting of specific receptors with a significant clinical application. Our data reveal when phage NL_ZS_3 resistance evolves, the resistant mutant strain of ZS15 showed increased tigecycline sensitivity, despite the mechanism underlying potential is not fully understood.

Tigecycline, known as a tetracycline antibiotic, has shown the greatest antimicrobial activity against CRKP. Genes e.g. *tet*(B), *tet*(A), *tet*(K), *tet*(M) and *tet*(S) coding for multidrug efflux pumps are membrane-related proteins and can efflux tetracyclines from the bacteria intracellularly, thus decreasing the MIC and subsequently allowing protein synthesis without affecting cell viability (Linkevicius et al., 2016). Using membrane-related proteins as a surface structure is one of the most common components functioning as a phage receptor. Therefore, it makes sense that phage-resistant defense strategies would confer a fitness cost for bacteria, i.e., becoming more sensitive to certain antibiotics. For instance, in *P. aeruginosa*, phage OMKO1 evolved to become less resistant to antibiotics. Likewise, in *E. coli*, the TolC protein evolved in a variety of diverse cellular functions, including antibiotic efflux and phage TLS receptor (Chan et al., 2016). However, unlike previous studies, the anti-phage TLS mediated TolC mutation did not influence its role in antibiotic efflux. Broadly, these results reveal that a phage-host arm race can result in different outcomes in terms of antibiotic susceptibilities, highlighting the diversity and complexity of host interactions within bacterial populations. Further, additional knowledge behind phage resistance in bacteria pathogens and the physiological consequences of acquiring phage resistance phenotypes mechanisms can be gained to renew the utility of ineffective antibiotics.

### Potential Applications of Phage Therapy

Successful application of phage therapy requires collaboration between microbiology and preclinical development; however, little is known about their alignment to guide future development. Since the discovery of phages, many have been isolated and identified as potential alternatives to antibiotics with different efficacy profiles. Phage specificity is both a benefit and an obstacle for clinical trials, as phage receptor diversity affects phage-host interaction. When the bacterial population is under phage attack, only a small fraction of cells can survive these selective pressures, and those that survive determine the fate of the population. Therefore, elucidating details of phage-host interactions and how these would influence bacterial physiological heterogeneity is fundamental to the development of phage therapy. In this study, the three highlighted phages and phage cocktail showed promising *in vitro* effects that perhaps these phages can be tamed for our benefits without negatively affecting their natural balance. The main limitation of this study is that only a small number of bacterial isolates and phages have been investigated. A large-scale study on the interactions between carbapenem-resistant *K pneumoniae* strains and phages covering a considerable spatial and temporal variability is essential for the successful application of phage-based pathogen control to fill our knowledge gap between *in vitro* and *in vivo* models.

## Data Availability Statement

The datasets presented in this study can be found in online repositories. The names of the repository/repositories and accession number(s) can be found in the article/supplementary material.

## Author Contributions

Conceptualization, DT and NL. Methodology, NL. Software, RB and YZ. Formal analysis, YZ. Investigation, NL and YZ. Resources, DT. Data curation, NL. Writing—original draft preparation, DT. Writing—review and editing, DT and BH. Supervision, DT and BH. Project administration, DT and BH. Funding acquisition, BH, TZ, and DT. All authors contributed to the article and approved the submitted version.

## Funding

This project was supported by the National Natural Science Foundation of China (NSFC) (No. 42006136) Shanghai Municipal Commission of Health (No.20204Y0336), Shanghai Public Health Clinical Center (KY-GW-2020-08), and Shanghai Pujiang Talents Plan Project (No. 2020PJ054) to DT and by the National Natural Science Foundation of China (NSFC) (No.82072325) and Clinical Research Plan of SHDC (No. SHDC2020CR2031B) to BH.

## Conflict of Interest

The authors declare that the research was conducted in the absence of any commercial or financial relationships that could be construed as a potential conflict of interest.

## Publisher’s Note

All claims expressed in this article are solely those of the authors and do not necessarily represent those of their affiliated organizations, or those of the publisher, the editors and the reviewers. Any product that may be evaluated in this article, or claim that may be made by its manufacturer, is not guaranteed or endorsed by the publisher.
